# Increasing Recognition of Community-Acquired, Non-Tuberculous Mycobacterial Infections of the Hand and Wrist

**DOI:** 10.7759/cureus.22105

**Published:** 2022-02-10

**Authors:** Jerec Ricci, Pierce Jones, Alice Le, Lisa L Steed, Milton B Armstrong, Fernando A Herrera

**Affiliations:** 1 Surgery, Medical University of South Carolina, Charleston, USA; 2 College of Medicine, Medical University of South Carolina, Charleston, USA; 3 Pathology and Laboratory Medicine, Medical University of South Carolina, Charleston, USA; 4 Plastic and Reconstructive Surgery, Medical University of South Carolina, Charleston, USA

**Keywords:** non-tuberculous mycobacteria, fishing injuries, plastic and reconstructive surgery, infectious disease pathology, hand surgery

## Abstract

Background

In this study, we present our experience with community-acquired, culture-positive, non-tuberculous mycobacterial (NTM) infections of the hand and wrist and compare the clinical features, risk factors, diagnostic delays, and treatment outcomes among patients referred for surgical consultation at our institution over a five-year period.

Methodology

We retrospectively identified patients on chart review who were diagnosed with culture-positive, extrapulmonary, cutaneous NTM infections between January 1, 2014, and December 31, 2018. Only patients with community-acquired NTM infections of the hand and wrist were included. Patient demographics, risk factors, location, diagnostic delays, NTM species isolated, treatment modalities, and treatment outcomes were collected and analyzed. These variables were further compared between patients who participated in fishing-related activities and those who did not.

Results

A total of 10 patients were identified with community-acquired NTM infections of the hand or wrist. Of these patients, eight (80%) were male, and six (60%) had participated in fishing-related activities prior to the initial presentation. The majority of patients had *Mycobacterium marinum* isolates (n = 6, 60%) and involved the hand (n = 8, 80%). *M. marinum* isolates were associated with a significantly shorter time to diagnosis (p = 0.02). All patients underwent surgical management with a prolonged course of postoperative antibiotics and were cured of their infection at the end of their treatment course.

Conclusions

Proper risk factor documentation and heightened clinical awareness are essential to reduce delays in the diagnosis of NTM skin and soft tissue infections and provide the best chance for curative therapy.

## Introduction

Non-tuberculous mycobacteria (NTM) are ubiquitously present in the environment and reside in many locations such as soil, biofilms, aerosols, and water. Over 200 species of NTM have been identified and are divided into rapid and slow-growing subtypes [1]. While NTM most commonly causes pulmonary disease, an increasing incidence of extrapulmonary cutaneous manifestations has been reported [2]. Although NTM comprises a relatively small percentage of the overall causes of skin and soft tissue infections, recent studies have found a nearly three-fold increase in the incidence of cutaneous NTM infections over the past few decades [3]. The skin and soft tissue infections caused by NTM are characterized by an indolent course and non-specific presentation, which often result in a delay in diagnosis three to five months after the first clinical signs appear [4]. Only after multiple diagnostic imaging studies and medical treatment failures are these infections routinely considered. In addition, exposure to aquatic environments and water-related activities is a well-known risk factor for cutaneous NTM infection, as reported by the majority of our patients in this study. These exposures need to be well documented to prevent further delays in diagnosis [4]. Surgical debridement is frequently required in addition to a combination of antibiotics to provide the highest chance of cure for cutaneous NTM [5,6]. Due to the rise in the incidence, unusual presentation, and a commonly delayed diagnosis, NTM should always be considered on the differential diagnosis in patients with an atypical infectious presentation, especially in the hand and/or wrist.

## Materials and methods

Study patients

Institutional review board approval was obtained prior to the initiation of this study. A retrospective review was performed to identify all patients who underwent debridement of the hand and/or wrist for a presumed infection between January 1, 2014, and December 31, 2018, had a positive NTM culture from surgical specimens, and had no history of upper extremity surgery or recent hospital admission. The following demographic information was extracted from the electronic health records of each patient: age, gender, date of initial presentation, date of surgery, date of culture positivity, comorbidities (obesity, body mass index (BMI), diabetes, hyperlipidemia, hypertension, immunosuppression), clinical presentation, exposure to known risk factors (water exposure, fishing, gardening), profession, duration of symptoms prior to presentation, time to diagnosis (time from initial presentation within the institution to established diagnosis from cultures), duration from exposure, time from surgical consultation to surgical intervention, diagnostic tests performed (e.g., including X-rays, electromyography (EMG), magnetic resonance imaging (MRI), acid-fast bacteria (AFB) operative cultures, antibiotic susceptibility testing), NTM species isolated, medical treatment regimens, and surgical procedures performed.

Patients’ disease was divided into complicated and uncomplicated NTM infections. Uncomplicated infections were defined as those confined to the skin or subcutaneous tissues during operative evaluation. Complicated infections were defined as those involving deeper tissue structures, such as tendons leading to tenosynovitis, during operative evaluation. No cases of osteomyelitis were identified in any of the patients.

Water exposure was defined as any documented contact with saltwater or freshwater and was further subdivided into patients who participated in fishing-related activities resulting in hand or wrist injury and those who did not.

Immunocompetent status was defined as patients having taken less than 10 mg prednisone daily for at least two weeks or equivalent [7] and those without any known pre-existing medical condition that could impair the immune response.

Patients were excluded from the study if their NTM specimen was isolated from a different anatomical location other than the hand and/or upper extremity, if no NTM specimens were isolated, or in those with an iatrogenic NTM etiology.

Statistical analysis

Statistical analysis was performed using GraphPad Software, version 8.0. Descriptive information regarding patients with NTM infections was reported as frequencies and proportions for categorical variables and mean ± standard deviation (SD) for continuous variables. P-values were reported, when appropriate, with statistical significance classified as p-values of <0.05.

## Results

Between January 1, 2014, and December 31, 2018, we identified 10 patients with a confirmed diagnosis of an NTM infection involving the hand or wrist who were referred for surgical consultation and eventually required surgical debridement. No patients in our cohort were identified to be immunocompromised. Most patients were male (n = 8, 80%) and had documented participation in fishing activities (n = 6, 60%) leading to hand or wrist injury (Table 1).

**Table 1:** Baseline patient demographics. SD = standard deviation; BMI = body mass index

Patient demographics	Count	%
Age (year), mean ± SD	57.7 ± 14.2	
Gender		
Male	8	80
Female	2	20
Comorbidities		
Obesity (BMI, kg/m^2^)		
20–24.9	4	40
25–29.99	4	40
>30	2	20
Diabetes	0	0
Hyperlipidemia	3	30
Hypertension	2	20
Immunosuppressed	0	0
Risk factors		
Water exposure		
Fishing	6	60
Contact with fish/shrimp	1	10
Boat cleaning	1	10
Gardening	1	10

Upon initial clinical presentation, the majority of patients reported pain (n = 7, 70%) and almost all patients had edema (n = 9, 90%), whereas relatively few patients had nodules (n = 3, 30%) or erythema (n = 3, 30%). Half of the patients had an open wound at the time of presentation. The majority of NTM infections involved the fingers (n = 6, 60%), were diagnosed via an AFB culture (n = 8, 80%) of tissue collected at the time of surgery, were found to have an NTM isolate of *M. marinum* (n = 6, 60%), and were complicated infections (n = 7, 70%) (Table 2).

**Table 2:** Clinical presentation and diagnosis. AFB = acid-fast bacilli; EMG = electromyography; MRI = magnetic resonance imaging; MAC = *Mycobacterium avium* complex; SD = standard deviation

Year of initial diagnosis	Count	%
2014	3	30
2015	3	30
2016	1	10
2017	2	20
2018	2	20
Presenting symptoms		
Pain	7	70
Edema	9	90
Nodules	3	30
Erythema	3	30
Open wound	5	50
Anatomical location		
Wrist	3	30
Finger	6	60
Thumb	1	10
Diagnostic tests		
AFB culture	8	80
Exudate culture	2	20
X-ray	6	60
EMG	2	20
MRI	5	50
Initial diagnosis		
Tenosynovitis	6	60
Skin and soft tissue infection	4	40
Carpal tunnel syndrome	2	20
NTM species isolated		
*M. abscessus*	1	10
MAC	3	30
*M. marinum*	5	50
Time from institutional presentation to diagnosis, months (mean ± SD)	3.0 ± 3.6	
Duration from exposure to diagnosis, months (mean ± SD)	5.0 ± 3.1	
Uncomplicated	3	30
Complicated	7	70

Following the initial presentation, most patients received a trial of preoperative management (n = 8, 80%), while only two (20%) patients went directly to surgical intervention. This was at the discretion of the surgeon based on the appearance of the wound and the likelihood of treatment failure with medical management. Half of all patients received a course of preoperative oral antibiotics and either one or two local steroid injections (Table 3). Steroid injections were only administered after no improvement with a trial of oral antibiotics prior to the investigation into atypical infections or operative intervention. All patients subsequently failed conservative management and underwent operative intervention. Furthermore, all patients received one of five different postoperative antibiotic regimens, all of which included a macrolide based on intraoperative NTM isolates, duration of symptoms, and severity of infection. *Mycobacterium avium* complex (MAC) infections were treated by adding ethambutol and rifampin to a macrolide, *M. abscessus* infections were treated using a combination of azithromycin with clofazimine, and *M. marinum* infections were treated with a macrolide and rifampin in addition to a third variable antibiotic. The most commonly prescribed postoperative antibiotic regimen was a combination of azithromycin, ethambutol, and rifampin (n = 4, 40%) for a mean duration of seven months (Table 3). At the end of postoperative antibiotic treatment, all patients were cured, as demonstrated by substantial clinical improvement, no recurrence of infection, and no further clinical signs or symptoms.

**Table 3:** Clinical and surgical management of non-tuberculous mycobacterial infections. TMP/SMX = trimethoprim/sulfamethoxazole; I&D = incision and debridement; SD = standard deviation; Azith = azithromycin; Etham = ethambutol; Rifamp = rifampin; Moxi = moxifloxacin; Clarith = clarithromycin; Doxy = doxycycline; Clofaz = clofazimine

Preoperative management						Number of patients			Percentage of patients		
Splints						3			30		
Antibiotics						5			50		
Doxycycline						2			20		
TMP/SMX						2			20		
Ciprofloxacin						1			10		
Local steroid injection						5			50		
X1 (right wrist, left middle finger, and left wrist						3			30		
X2 (right index finger, left index finger)						2			20		
Oral steroids						2			20		
Bedside I&D						1			10		
Operative management						10			100		
Radical synovectomy						2			20		
Radical tenosynovectomy						2			20		
Tenosynovectomy						4			40		
Carpal tunnel release						3			30		
Incision and drainage						4			40		
Number of operative interventions						Mean ± SD = 1.4 ± 0.8					
Postoperative antibiotic regimens											
Regimen number	Number of patients (%)	Azith	Etham	Rifamp			Moxi	Clarith		Doxy	Clofaz
1	4 (40)	x	x	x							
2	3 (30)		x	x				x			
3	1 (10)	x		x						x	
4	1 (10)	x		x			x				
5	1 (10)	x									x
Length of postoperative antibiotic treatment, months (mean ± SD)					7.0 ± 2.7						

While antibiotic susceptibilities for *M. marinum* have been well described and acquired resistance is rare [4], antibiotic susceptibilities for MAC and *M. abscessus* are more variable. Therefore, we only conducted antibiotic susceptibility testing for MAC and *M. abscessus* species. This allowed for antibiotic regimens to be individually tailored for those with MAC or *M. abscessus* isolates.

Due to the high incidence of fishing-related water exposure leading to an injury of the hand or wrist, we performed a further subanalysis to compare patients participating in fishing-related activities and those who did not prior to presentation. When including all combined cases involving the hand and wrist, we found that there was no significant difference in age, time to diagnosis, the number of surgical debridements, length of postoperative antibiotic treatment, or time from initial surgical consultation to surgical intervention between those who participated in fishing-related activities and those who did not (Table 4). However, after only including patients with injuries to their hand (finger, n = 6, or thumb, n = 1), we discovered that there was a significantly shorter time to diagnosis (p = 0.001) and time from surgical consultation to surgical intervention (p = 0.004) in patients who had well-documented participation in fishing-related activities compared to those who did not. Only one patient with an NTM infection of the wrist was found to have an *M. marinum* isolate, while the other two patients with NTM infections of the wrist were found to have MAC isolates. Interestingly, all patients with MAC or *M. abscessus* isolates had a significantly longer time to diagnosis than those with isolates of *M. marinum* (p = 0.02) (Table 4).

**Table 4:** Non-tuberculous mycobacterial infections related to fishing activities in all hand and wrist cases or hand cases only. SD = standard deviation; MAC = *Mycobacterium avium* complex

NTM infections in the hand and wrist (n = 10)					
	Fishing		No fishing		P-value
Age, years (mean ± SD)	58.3 ± 17.4		60.0 ± 10.1		0.87
	Count	%	Count	%	
Male	6	60	2	20	
Female	0	0	2	20	
NTM species isolated					
M. abscessus	0	0	1	10	
MAC	2	20	1	10	
M. marinum	4	40	2	20	
Time to diagnosis, months (mean ± SD)	2.8 ± 4.6		3.3 ± 1.7		0.86
Number of surgical debridements (mean ± SD)	1.3 ± 0.8		1.5 ± 1.0		0.78
Length of postoperative antibiotic treatment, months (mean ± SD)	7.7 ± 3.4		6.0 ± 0.0		0.37
Time from initial surgical consultation to surgical intervention, months (mean ± SD)	2.3 ± 3.6		3.3 ± 1.8		0.65
NTM infections in the hand (n = 7)					
	Fishing		No fishing		P-value
Age, years (mean ± SD)	52.3 ± 17.9		55 ± 1.7		0.81
	Count	%	Count	%	
Male	4	57	1	14	
Female	0	0	2	29	
NTM species isolated					
*M. abscessus*	0	0	1	14	
MAC	0	0	1	14	
*M. marinum*	4	57	1	14	
Time to diagnosis, months (mean ± SD)	0.5 ± 0.3		4.0 ± 1.0		0.001
Number of surgical debridements (mean ± SD)	1.0 ± 0.0		1.7 ± 1.15		0.29
Length of postoperative antibiotic treatment, months (mean ± SD)	5.5 ± 1.0		6.0 ± 0.0		0.44
Time from initial surgical consultation to surgical intervention, months (mean ± SD)	0.6 ± 0.3		4.0 ± 1.3		0.004
NTM infections by isolate (n = 10)					
	*M. marinum* (n = 6)	MAC/*M. abscessus* (n = 4)			P-value
Time to diagnosis from initial institution presentation, months (mean ± SD)	1.0 ± 1.0	6.0 ± 4.1			0.02
Time from exposure to presentation at institution, months (mean ± SD)	2.3 ± 2.3	1.5 ± 1.3			0.39

## Discussion

Despite multiple studies detailing the increasing incidence of extrapulmonary cutaneous NTM infections over the past few decades [3,8], these infections continue to present with an atypical clinical presentation and may result in delays in care. While there have been reports of delay in diagnosis of up to 180 months [9], a recent study involving 44 patients with cutaneous NTM infections reported a diagnostic delay of ≤36 months. The study further identified that diagnostic delays of more than or equal to four months were associated with worse outcomes [1]. In our study, no patient had a diagnostic delay of more than 12 months, and we did not observe a difference in outcome with the number of surgical debridements or the length of postoperative antibiotic treatment. All patients were successfully cured at the completion of their treatment course using a combination of surgical intervention and postoperative antibiotics. However, our findings highlight that fishing exposures along with MAC or *M. abscessus* NTM isolates may result in delayed diagnosis regarding NTM infections related to the hand and wrist.

Cutaneous NTM infections involving the hand and wrist have been increasingly recognized by others in case reports and single-center studies [3,10-16]. While there has been speculation regarding the explanation for these observations [3], the etiology for this increase remains unclear. However, it is well known that cutaneous NTM infections are associated with surgical procedures, traumatic injuries or puncture wounds, and activities such as fishing, gardening, farming, and aquarium handling [17]. Risk factors also correlate closely with the NTM species identified [8]. *M. marinum* was the most commonly reported organism in NTM infections of the upper extremity [3,4,17], which is consistent with our findings. *M. marinum*, in particular, is found in aquatic environments of freshwater and saltwater and is isolated from marine animals [18]. Although *M. marinum* is the most well-described NTM species associated with aquatic environments, *M. abscessus* is also known to be related to water exposure [1]. Classically, *M. marinum* infections of the hand are associated with marine trauma in the immunocompromised [15]. While none of the patients in our study were found to be immunocompromised, 60% were found to have *M. marinum* isolates, and 83% of those patients had documented aquatic exposure. This is slightly higher than the 69% with aquatic exposure reported by Hess et al. in a larger study of 29 patients with upper extremity infections secondary to *M. marinum* [15]. While this study did not further delineate the types of aquatic exposure, 66% of our patients with *M. marinum* isolates had participated in fishing-related activities. This is in contrast to a larger study of 55 patients with upper extremity *M. marinum* infections out of Denmark that observed a much higher association with exposure to fish tanks (83%) compared to fishing (5.7%) [18].

Maintaining awareness of specific associated risk factors in patients who present with atypical infections of the hand and wrist may help reduce the delays in diagnosis [4]. According to our findings, patients who had a well-documented exposure to fishing-related activities were more likely to have a shorter time to diagnosis and shorter time from initial surgical consultation to surgical intervention when the NTM infection involved the hand. Furthermore, those who were found to have *M. marinum* isolates had a shorter time to diagnosis than those with either MAC or *M. abscessus* isolates. However, a larger study of 34 patients with NTM infections of the upper extremity by Lopez et al. reported a significantly longer time to diagnosis with *M. marinum* species [16]. Interestingly, the majority of patients included in this study had unknown exposures, with only five of 34 patients having an identifiable aquatic exposure. Although documentation of risk factors is important in reducing time to diagnosis, it has been reported that up to 31% of patients may not recall a water-related activity or associated traumatic puncture wound due to the prolonged incubation period of this organism, which may result in further delays in care [15]. Therefore, other diagnostic modalities with the potential to generate faster results may prove to be beneficial in reducing delays in care.

While tissue biopsy and culture remain the gold standard for diagnosis, NTM cultures in solid agar require long incubation times, ranging from seven days for rapidly growing species to over three weeks for slower-growing species [2]. Additionally, AFB cultures are typically only positive in 70-80% of cases [15]. This has led to the investigation of DNA probes, broad-range and targeted PCR assays sequencing 16S ribosomal RNA (rRNA) or hsp65, respectively, and matrix-assisted laser desorption ionization-time of flight mass spectrometry (MALDI-TOF-MS) that may expedite the process [2,19]. Another strategy employed by Lopez et al. was to routinely include lab orders and cutaneous biopsy specimens for patients who presented with an atypical upper extremity infection or who failed antibiotics for common pathogens [16]. While this protocol may have facilitated a more timely diagnosis, an increase in cases was also observed following its implementation suggesting that NTM cases may have previously been underreported. From our experience, MALDI-TOF-MS was the most commonly used confirmatory test, although this testing modality may have utility as an initial diagnostic test to identify the presence of AFB within a period as short as one day compared to at least seven days for rapidly growing NTM in solid agar [2].

Similar to other studies [3], NTM infections in our study are likely underreported because patients were required to have a positive AFB culture to be included. Other limitations include the population, which represents mostly patients who live and work in the coastal Carolina area and may not be generalizable to other regions. Finally, due to the retrospective nature of this study, we relied on accurate documentation from the electronic medical record.

## Conclusions

Compared to other possible pathogens, NTM is a relatively uncommon cause of cutaneous infections in the hand and wrist; however, reducing delays in diagnosis is key to their successful management. In those who have failed conservative management, increased clinical suspicion, risk factor documentation, and a lower threshold for diagnostic testing are essential for the successful identification and treatment of NTM infections of the hand and wrist. Moreover, a combination of surgery and postoperative antibiotic therapy is often required to provide the best chance for curative treatment of NTM infections of the hand and wrist.
